# Current Projection Methods-Induced Biases at Subgroup Detection for Machine-Learning Based Data-Analysis of Biomedical Data

**DOI:** 10.3390/ijms21010079

**Published:** 2019-12-20

**Authors:** Jörn Lötsch, Alfred Ultsch

**Affiliations:** 1 Institute of Clinical Pharmacology, Goethe-University, Theodor-Stern-Kai 7, 60590 Frankfurt am Main, Germany; 2 Fraunhofer Institute for Molecular Biology and Applied Ecology IME, Project Group Translational Medicine and Pharmacology TMP, Theodor-Stern-Kai 7, 60590 Frankfurt am Main, Germany; 3 DataBionics Research Group, University of Marburg, Hans-Meerwein-Straße, 35032 Marburg, Germany; ultsch@informatik.uni-marburg.de

**Keywords:** flow cytometry, high-dimensional data sets, computational techniques, machine-learning, data science, t-distributed stochastic neighbor embedding, emergent self-organizing maps, immunological research

## Abstract

Advances in flow cytometry enable the acquisition of large and high-dimensional data sets per patient. Novel computational techniques allow the visualization of structures in these data and, finally, the identification of relevant subgroups. Correct data visualizations and projections from the high-dimensional space to the visualization plane require the correct representation of the structures in the data. This work shows that frequently used techniques are unreliable in this respect. One of the most important methods for data projection in this area is the t-distributed stochastic neighbor embedding (t-SNE). We analyzed its performance on artificial and real biomedical data sets. t-SNE introduced a cluster structure for homogeneously distributed data that did not contain any subgroup structure. In other data sets, t-SNE occasionally suggested the wrong number of subgroups or projected data points belonging to different subgroups, as if belonging to the same subgroup. As an alternative approach, emergent self-organizing maps (ESOM) were used in combination with U-matrix methods. This approach allowed the correct identification of homogeneous data while in sets containing distance or density-based subgroups structures; the number of subgroups and data point assignments were correctly displayed. The results highlight possible pitfalls in the use of a currently widely applied algorithmic technique for the detection of subgroups in high dimensional cytometric data and suggest a robust alternative.

## 1. Introduction

Recent advances in flow cytometry and other molecular laboratory techniques allow the acquisition of large and high-dimensional data sets. These developments have been accompanied by the implementation of new computational techniques allowing the visualization and analysis of the acquired data and, finally, the identification of relevant subgroups in data sets [1]. Computational flow cytometry has been identified as an important new field at the interface of immunology and computational biology, enabling new biological knowledge to be gained from cell-based high-throughput data [1].

The main goal of computational analyses of high-dimensional biomedical data is the identification of relevant subgroups of patients or cell populations. Therefore, it is crucial that the computational methods used perform this task correctly. This requires distance-preserving data projection techniques that neither impose non-existing structures on data nor interfere with existing data structures. Therefore, we have analyzed data projection techniques that are currently widely used for this purpose, in particular, t-distributed stochastic neighborhood embedding (t-SNE [2]). t-SNE is increasingly used in the workflows of biomedical research. A PubMed search (https://www.ncbi.nlm.nih.gov/pubmed) for “(t-sne or “t-distributed stochastic neighbor embedding” or “t-statistic stochastic neighbor embedding”) NOT review” performed on 25 October 2019 resulted in 191 hits, with an increase in publication numbers per year from two in 2010 to 88 in 2019.

**Figure 1:**
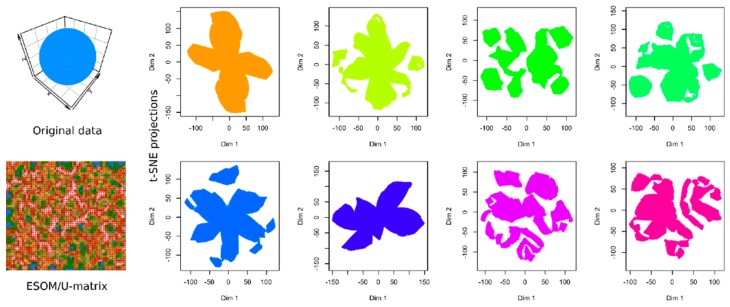
Results of t-distributed stochastic neighborhood embedding (t-SNE) analyses (eight panels in columns 2–5) applied on a cluster-free artificial data set #1 (1st panel in the upper left corner, data set #1). The data set is composed of 4002 data points merely arranged on the surface of a sphere at equal distances. Results of an alternative projection and subgroup detection technique, implemented as ESOM/U-matrix, which clearly indicate the absence of any systematic data structures, are shown at lower-left panel. The figure has been created based on the t-SNE analysis implemented in the R library “tsne” [3] and the U-matrix was obtained using the R library “Umatrix” [4].

To illustrate the relevance of evaluating the performance of the currently recommended computational techniques in data projection, Figure 1 shows the results of a t-SNE analysis of a structure-less artificial data set (upper left panel). No special hyperparameter adjustment was made for the algorithm because these analyses are often performed by domain experts, such as clinical researchers, who use the default parameters implemented in the software. The data set consists of 4002 data points spaced equally on the surface of a sphere. Nowhere does it contain any of the groups suggested by the results of some of the t-SNE analyses presented in the subsequent panels of Figure 1. In fact, multiple use of the same t-SNE algorithm produced different results that occasionally trigger interpretations of different numbers of subgroups in the data set.

This example shows that the uncritical use of computational techniques is a pitfall of biomedical research that leads to data analysis-based biases due to unobserved problems in data transformation and projection. This is particularly true in environments where these techniques are implemented in standard software packages bundled with laboratory equipment to cover the entire data acquisition and analysis workflow.

With an emphasis on t-SNE as a data projection technique that appears to be becoming a standard in flow cytometry, this paper, therefore, aims to evaluate the results of this technique when applied on several different artificial or biomedical data sets. To demonstrate that data analysis is not limited to a single method that is occasionally delivered with the laboratory device, an alternative approach has been used in parallel to t-SNE, consisting of emergent self-organizing feature maps (ESOM) that have been shown to reliably detect structures in artificial and biomedical data sets [4,5]. The following report will identify potential pitfalls in the use of t-SNE for the analysis of data in molecular research and provide some technical background to these shortcomings; however, it cannot substitute the fundamentals of data science and tuning of analytical methods, which requires further reading presented in a broader context such as [6]. In this respect, the report emphasizes the need for collaboration between topical experts in biomedical research and methodological experts in data science.

## 2. Results and Discussion

### 2.1. Results of t-SNE Analysis in Artificial Data Sets

The results of the t-SNE projection of data set #1, i.e., the structure-less “golf ball” shaped data set, were presented as an introductory example in Figure 1. The eight analyses shown in Figure 1 were performed identically, with the exception that different “seed” values were used for the t-SNE runs. In some of the individual t-SNE analyses, there were clearly separated regions of data points that required the interpretation as a subgroup structure. However, they were produced by the t-SNE based projection of the data and do not reflect real data structures. Thus, t-SNE seems to provide different results depending on the random circumstances of the analyses. This is probably due to the fact that, as explained in the methods section, minimizing the Kullback–Leibler divergence during t-SNE is done with a gradient-descent method that is known to end in local minima. In addition, t-SNE is a probabilistic projection method, which means that it can lead to fundamentally different solutions depending on the initial randomly chosen output vectors (Figure 1).

**Figure 2:**
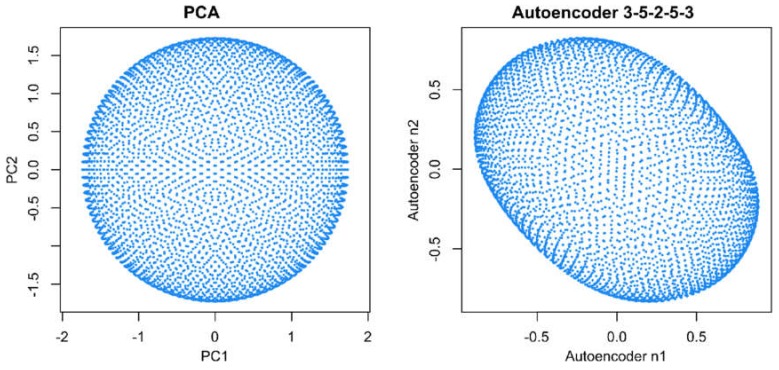
Visualization of projections of data set #1 (“golf ball data”) onto the two-dimensional projection space, ${\mathbb{R}}^{2}$, using either principal component analysis (PCA, left panel) or an autoencoding neuronal network (right panel). Both methods indicate the absence of a subgroup structure. The figure has been created using the R libraries “FactoMineR” [8] and “ANN2” [9].

When analyzing the same data using emergent self-organizing feature maps (ESOM), projecting the data on a grid of thousands of artificial neurons [7] and combining it with U-matrix methods [4,5], it was correctly concluded that there is no group structure in these data. As shown in the lower-left panel of Figure 1, the U-matrix consists of a random structure without subgroups. The present result is similar to that of a previous analysis, which was interpreted similarly as indicating the absence of any subgroup structure in this data set (see Figure 3 in [5]). To further reinforce that the data set does not have a subgroup structure, two additional data projection techniques were applied, which include (i) principal component analysis and (ii) auto-encoding neuronal networks (Figure 2). Similar to the results of the ESOM/U-matrix and in contrast to the results of t-SNE, it was clearly indicated that the data set does not contain a subgroup structure.

**Figure 3:**
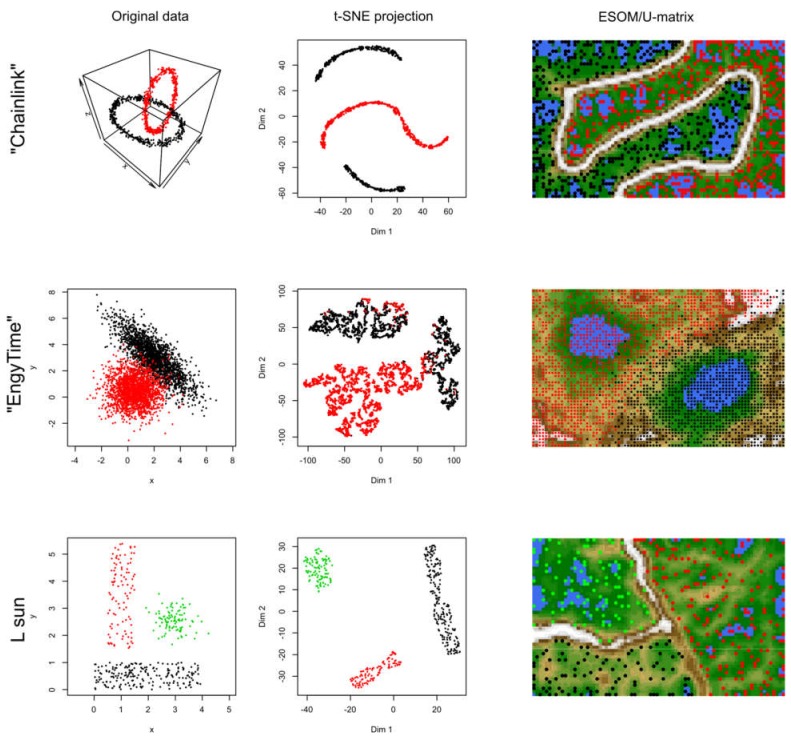
Results of t-SNE analyses applied to artificial data sets #2–#4 containing one or two clusters (see color code). The original data sets are shown in the left panels, the projections obtained with t-SNE in the corresponding middle panels. An alternative projection and subgroup detection technique, implemented as ESOM/U-matrix, is shown at the right panels. The figure has been created based on the t-SNE analysis implemented in the R library “tsne” [3]) and the U-matrix obtained using the R library “Umatrix” [4].

In data sets #2 to #4 (Figure 3), the performance of t-SNE was heterogeneous. The “Chainlink” data set, which contains two clearly visible subgroups arranged as interconnected rings, was projected by t-SNE in a way that indicated three clusters (Figure 3). The “EngyTime” data set, which contains a circular and an elliptic subgroup with a wide transition zone between them where the cluster membership is visible by differences in data density (Figure 3), was projected to actually display the two clusters. However, errors were made in the data point association to the correct subgroup, which are visible in the “wrong” coloration of the t-SNE projection-based groups. For the “Lsun” data set, which consists of three clusters arranged at clear distances from each other, t-SNE provided the correct results in terms of subgroup number and data point allocation (Figure 3). Thus, t-SNE seems to perform well when the data subgroups are clearly separated and spaced. In contrast, for clusters that can be separated by data point density and not by data point spacing, as with “EngyTime”, t-SNE is prone to errors.

In contrast to t-SNE, the results obtained using the ESOM/U*-matrix approach were always correct (right column of panels in Figure 3). This method not only captures clusters separated by distances but also by data density, which explains why, in such situations, it outperforms t-SNE, which concentrates only on the first cluster property.

### 2.2. Results of t-SNE Analysis in Biomedical Data Sets

In data sets #5 and #6, which originate from or are similar to flow-cytometric experiments, the performance of t-SNE was again heterogeneous.

**Figure 4:**
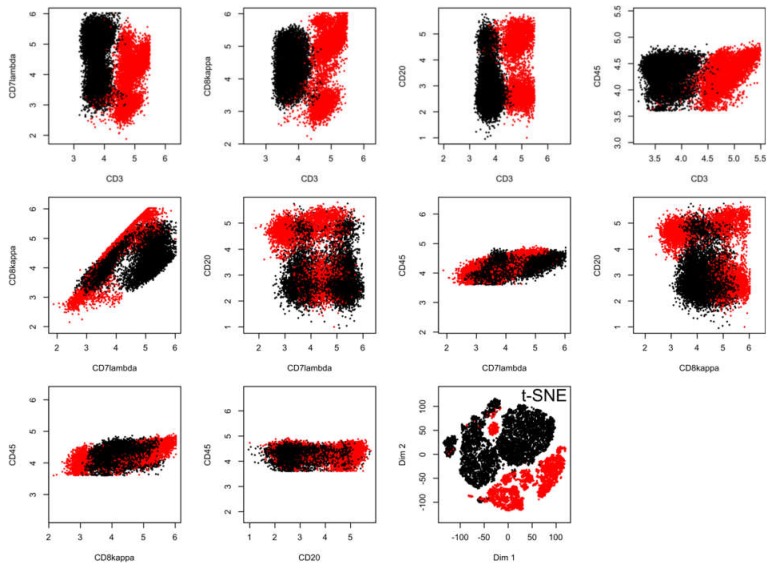
Results of a t-SNE analysis applied on a FACS data set from an analysis of different cell surface markers in chronic lymphocytic leukemia (CLL) patient data versus healthy controls (data set #5). The first ten panels, starting from the upper left corner, display the original data, each marker against all others in two-dimensional plots. The projections obtained with t-SNE are shown at the last panel on the right side of the lower line of panels. Please see Figure 6 for an alternative projection technique. The figure has been created based on the t-SNE analysis implemented in the R library “tsne” [3]).

In the flow-cytometric data set #5, which comprised 18558 data points including expression of CD3, CD7lambda, CD8kappa, CD20, and CD45, acquired from n = 14 healthy subjects and n = 10 patients with chronic lymphocytic leukemia (Figure 4), the t-SNE data projection suggested several groups while the true two-group structure in the results is barely visible even with the corresponding staining of the projected data points (lower right panel of Figure 4). In addition, the allocation of data points reflecting the two different clinical diagnoses was incorrect if a small cluster of data points at the top of the projection was considered a separate group, as this cluster consisted of data points belonging to a group, a healthy or CLL patient.

**Figure 5:**
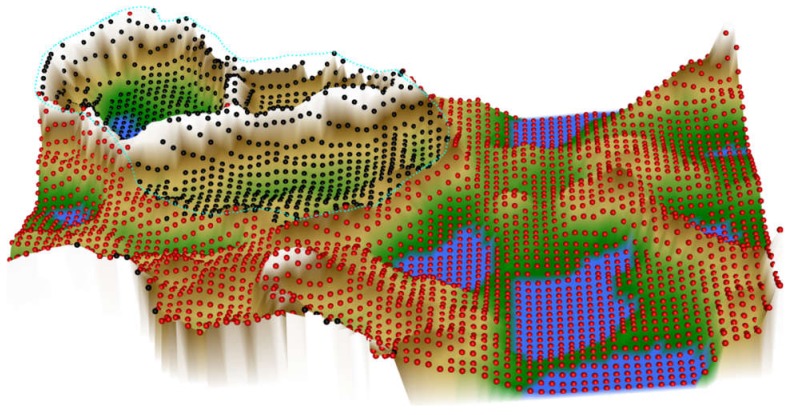
Results of an ESOM/U*-matrix analysis performed on the same data set #5 shown in Figure 4. A subgroup (marked with black dots in the figure) is surrounded on the U*-matrix by walls indicated with a white color, which corresponds to large distances in the data. This separates this group clearly from the rest of the cohort, marked with red dots in the figure. To show this separation, the cutting line of the U-matrix island was placed in a way that emphasizes group separation. The black-marked group in itself, however, might contain further subgroups, as indicated by the large mountain range crossing it. For the given data, one can assert that the red and black marked groups are distinct and that the inner variance of the black group is larger than within the red group. The figure has been created using the R library “Umatrix” [4].

In the ESOM/U*-matrix based data projection (Figure 5), the two-subgroup structure was mapped better than in the t-SNE based projection. Data from the same diagnostic subgroup were projected in close proximity, and the groups were separated by the structures of the U-matrix. However, the ESOM/U-matrix projection also provided indications that the group might be more complex, i.e., the CLL data might contain further subgroups of patients, as shown by the large mountain range that crosses its projection area.

**Figure 6:**
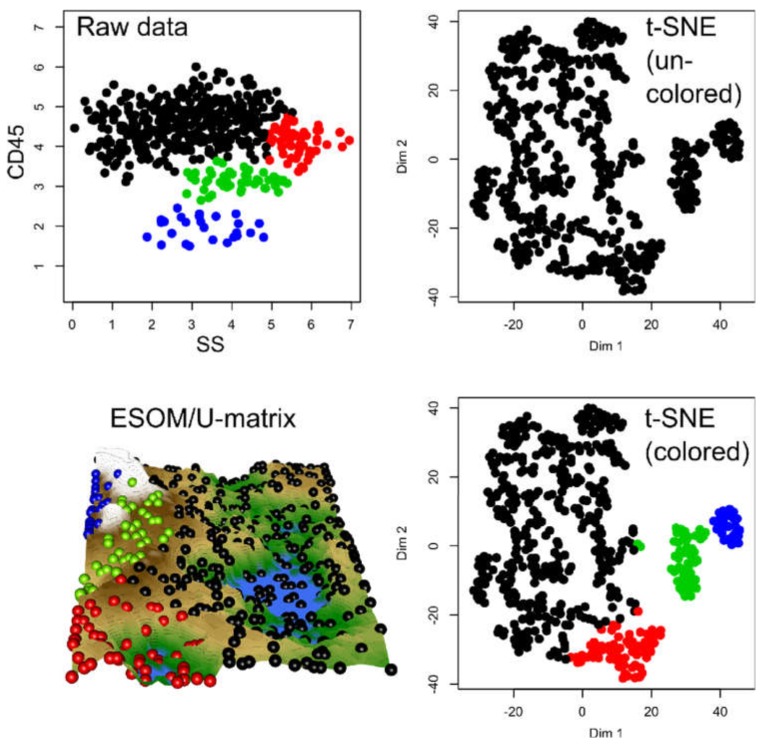
Results of a t-SNE analysis applied to data generated by a Gaussian mixture model with 4 modes derived from a FACS dataset (data set #6). The first panel in the upper left corner displays the original data. The projection obtained with t-SNE is shown at the second (not colored) and 4th (colored) panels. An alternative projection and subgroup detection technique, implemented as ESOM/U-matrix, is shown at the bottom left panel. The figure has been created based on the t-SNE analysis implemented in the R library “tsne” [3] and the U*-matrix was obtained using the R library “Umatrix” [4].

Finally, the t-SNE projection of data set #6 (Figure 6), which was derived from a true FACS data set, although modified with a Gaussian mixture model with 4 modes, indicated a subgroup structure (Figure 6). However, only two or three groups were suggested. Only when coloring the groups, the original four-group structure became visible.

### 2.3. Causes of Heterogeneous t-SNE Performance in Different Data Sets

The identification of subgroups or clusters is a key objective in the analysis of high-dimensional biomedical data [10]. Since the high-dimensional data space is not readily accessible for interpretation, the data must be projected onto a lower-dimensional space, often a two-dimensional space, which can then be investigated for subgroup or cluster structures. Several different data projection methods have been introduced but it is crucial that the projection methods maintain the topology of the data, i.e., ensure that data separated by long distances in the high dimensional space remain separated by large distances even after the data set has been projected onto low dimensional space.

Projections from high dimensions to low dimensions can be assessed taking into account two types of errors: forward projection errors (“trustworthiness”) and backward projection errors (“continuity”) [11]. By using the asymmetric version of the Kullback–Leibler divergence (KD; [12]), only one error direction is highlighted by t-SNE and thus, the reliability of the resulting projection is questionable. The “clusters” represented by a t-SNE projection are strongly influenced by the parameterization of the algorithm. It can be doubted that people other than the developers of t-SNE have enough understanding to always set these parameters correctly. This becomes even more relevant in the common laboratory environment where the data is analyzed by biomedical scientists and not by data scientists, using the software solutions implemented in the laboratory equipment by trying to cover the full workflow.

The present analyses show that the default settings of parameters probably indicate subgroups that are not in the data but result from the data projection performed by t-SNE. The central problem is that the Euclidean distance seen when viewing the two-dimensional output plane does not correctly represent the distances of the data in high-dimensional space. All projections from the high dimensional space R^D^ to lower dimensions R^d^, with d << D, must make errors because the high dimensional space simply does not fit into the low dimensional space.

In contrast to other methods such as t-SNE, the (generalized) U-matrix regards these errors. In fact, this method displays the errors of any projection [13] and shows them as a third dimension (“hill”) on the output plane. Therefore, ESOM/U-matrix based methods credibly represent the data space and avoid the pitfalls of spurious clusters.

### 2.4. Effects of Tuning the t-SNE Performance

The t-SNE analyses and the ESOM/U-matrix analyses had been performed using the default settings of the respective R libraries. This is a common case in research environments where data analysis is performed by topical scientists who use software packages where the defaults of the hyperparameters are preset to cover a broad variety of data problems. However, it is also well-known that tuning hyperparameters of algorithms can improve their performance.

**Figure 7:**
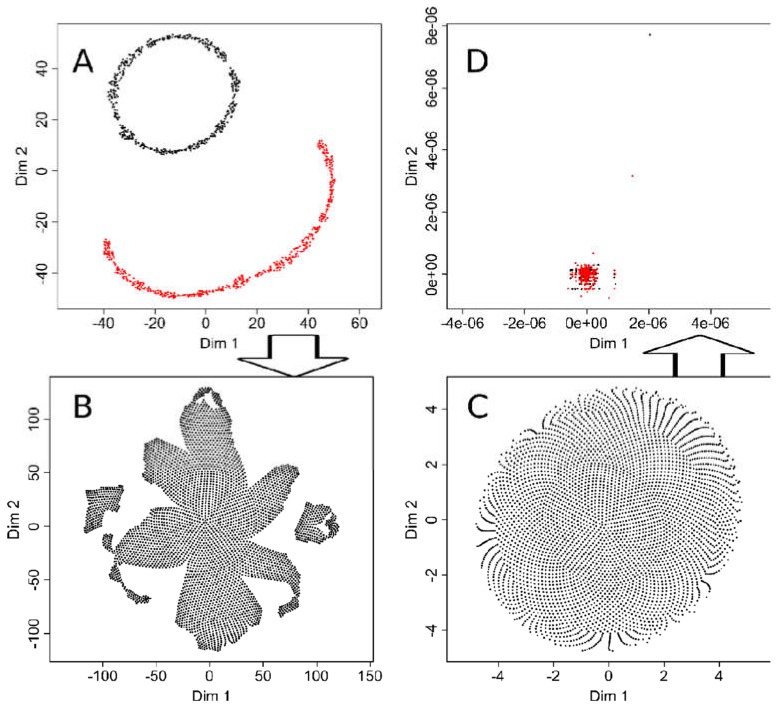
Effects of tuning of t-SNE hyperparameters. (**A**): t-SNE projection of the “ChainLink data set #2 with hyperparameters of the R library “tsne” [3] tuned to enhance the separation into two groups. The result is correct, whereas without tuning, the result had suggested three groups (Figure 2 upper middle panel). (**B**): When using t-SNE with the thus tuned hyperparameters on the “golfball” data set #1, the result suggested a group structure as obtained with the default parameters of the “tsne” library. (**C**): Vice versa, when tuning the t-SNE hyperparameters for the “golfball” data set, the result correctly indicated no structure in the data. (**D**): However, using the thus tuned t-SNE on the “ChainLink” data set failed to produce the expected result.

To estimate the possible consequences of hyperparameter tuning, t-SNE analyses of data sets #1 and #2 were repeated, while the ESOM/U-matrix had always worked correctly and therefore required no further adjustments. In particular, the parameters “initial_dims”, which controls the number of dimensions to use in reduction method [3], and in particular “perplexity”, which defines the optimal number of neighbors to be considered in the data projection [3], were tuned to the correct result for the “ChainLink” data set #2. This means that the projection now clearly showed two subgroups in the data set (Figure 7A). When using the same hyperparameter values in a t-SNE analysis of the “goofball” data set #1; however, the tuning had no effect. Here too, at least three subgroups were suggested by the projection of the structureless data set (Figure 7B).

Certainly, t-SNE could also be tuned to obtain the correct result with the “golfball” data set, i.e., when the “perplexity” parameter was set to a high value of 2000 (Figure 7). However, the projection of the two-group “ChainLink” data set then became incorrect (Figure 7D). Hyperparameter tuning thus increases the performance of t-SNE, but it seems difficult to find adjustments that work on a completely different data set. So for effective tuning, the results must be known, which calls into question the use of this analytical method to find structures in unknown data sets. Therefore, demonstrations of results of t-SNE based on the default settings of the software implementations seem justified to point to related pitfalls in biomedical research.

## 3. Materials and Methods

### 3.1. Data Sets

To assess the results of t-SNE in terms of data projection and subgroup visualization, artificial and biomedical data sets were selected for which the subgroup structures and the assignment of each data point to a particular subgroup were known.

The 1st data set consisted of the “golf ball” shaped data set, which served as an introductory example. It consists of 4002 data points located on the surface of a sphere. Each data point was placed at equal distances from six nearest neighbors.

The 2nd to 4th data sets came from an available collection of artificial data sets created for benchmarking of cluster- and data projection algorithms [14]. The data sets are available within the “Fundamental Clustering Problems Suite (FCPS)” at https://www.uni-marburg.de/fb12/arbeitsgruppen/datenbionik/data?set_language=en [14]. The data sets in FCPS are specifically designed to test the performance of projection and clustering algorithms for specific challenges, e.g., outliers or data-density versus data-distance defined clusters. This is typically represented by the three data sets selected for the present evaluations, which are included in the FCPS under the names “Chainlink”, “EngyTime”, and “Lsun” (Figure 3).

The 5th data set comprised biomedical data from a flow-cytometric experiment on the expression of cell surface molecules of the cluster of discrimination (CD) type. It had been assessed in white blood cells of patients with different types of lymphoma. In particular, the data set contains biomedical flow-cytometric data in which the expression of six different “clusters of discrimination” (CD) related proteins, found on the surface of cells, were analyzed, i.e., CD3, CD7lambda, CD8kappa, CD20, and CD45. Samples were taken from *n* =14 healthy subjects and *n* = 10 patients with chronic lymphocytic leukemia (CLL). In the present calculations, *n*_1_= 12,683 data points from healthy patients and *n*_2_ = 5875 data points from CLL patients were used. The present data set was obtained by uniform random sampling from a larger data set [15], using a gate for the selection of B-cells.

The 6th data set consisted of data derived from flow-cytometry modified with a Gaussian mixture model (GMM) containing four distinct modes. This data set was modeled on real FACS data from lymphoma diseases [16]. They were obtained in a flow cytometric analysis of the expression of cell surface molecules of the cluster of discrimination (CD) type, assessed in the white blood cells of patients with different types of lymphomas. The data set included two measurements that quantified SS (side scatter) and CD45 expression.

### 3.2. Data Projection and Subgroup Identification Using t-SNE

Data were analyzed using the R software package (version 3.6.1 for Linux; http://CRAN.R-project.org/; [17]) on an Intel Core i9® computer running Ubuntu (Linux 18.04.3 LTS 64-bit). The analyses were performed using a standard R implementation of t-distributed stochastic neighbor embedding (t-SNE [2]) available in the library “tsne” (https://cran.r-project.org/package=tsne [3]). The default settings of the t-SNE parameters implemented in the R library were used, as this is a common setting in laboratory research where the analyses are performed by the biomedical scientists using the software provided with the laboratory equipment.

t-SNE belongs to the family of focusing projections, i.e., it uses a notion of data point neighborhood *N*_D_ of a point in the high dimensional (*R^D^*) input space, and a neighborhood *N_d_* in the (*R*^d^) *d* = 2-dimensional output space (projection plane). In principle, t-SNE follows the idea that was originally put forward by Kohonen in his feature maps of artificial neurons [18]. There, the locations of neighbors in the input space were to be preserved on the projection plane i.e. *N_d_ ~ N_D_*. For this preservation, t-SNE minimizes the non-symmetric Kullback–Leibler divergence (KD; [12]) between the probability distribution *p_D_* of *N_D_* and the probability distribution *p_d_* of *N_d_*:

(1) $$KD\left(p_{D},p_{d}\right)=\int p_{D}*\frac{p_{D}}{p_{d}}$$

The probability for *p_D_* is estimated for pairs of data points *xi*, *xj* having an Euclidean distance *D(xi,xj)* via a Gaussian model for the high dimensional neighborhood *N_D_ ~ N(D(xi,xj), xi,s)* for some (critical) regularization parameters. The probability *p_d_* for the neighborhood *N_d_(d(xi,xj))* in the output plane with data distances *d(xi,xj)* is estimated using a t-distribution with one degree of freedom. The t-distribution has been chosen used for its heavy tail, after noting that Gaussians in the output space would not consider larger distances *d(xi,xj)*>>0.

### 3.3. Data Projection and Subgroup Identification Using ESOM

As an alternative projection method, emergent self-organizing feature maps (ESOM) were used, which project the data on a grid of thousands of artificial neurons [7]. In combination with U-matrix methods, it has been shown that structures are effectively detected in artificial and biomedical data sets [4,5]. These computational methods were performed using the R-library “Umatrix” (https://cran.r-project.org/package = Umatrix; [4]). Again, the defaults were used, such as a size of the projection plane of 50 × 80 neurons or a Gauss-formed neighborhood function and the use of 20 training epochs for the SOM.

As mentioned previously [19], one feature of this SOM usage is the large number of neurons, unlike the other prototype of SOMs, which is also used in common methods applied to flow cytometric data such as FlowSOM [20], where neurons are identified with clusters and, therefore, limited to a small number. In ESOM, emergence, i.e., the appearance of higher-level structures due to micro-scale interactions can be observed by looking at structures like ridges or valleys consisting of groups of neurons [21]. Learning in ESOM is performed using the SOM learning rule:

(2) $$\Delta w_{i}=\eta \left(t\right)h\left(bmu_{i,},r,t\right)\left(x_{i}-w_{i}\right)$$

with*x_i_* representing a data point, *bmu_i_* the neuron which is closet to *x_i_* in the SOM (best matching unit), *w_i_* the high dimensional representation vector of neuron *n_i_, h(…)* a neighborhood function and as learning rate $\eta \left(t\right)\in \left[0,1\right]$. During the training phase of ESOM, both the learning rate and neighborhood are decreased [15].

The U-matrix visualizes the structures of the distances in the high dimensional data space on top of the neuronal grid of the ESOM. For a neuron *n_i_*, a single point U-height is calculated as the sum of the data distances *d(w(n_i_), w(n))* to the immediate neighbors *n* of *n_i_* in the grid of neurons. The visualization facilitates the identification of subgroups by displaying the distances between the neurons in the high-dimensional space in a color-coding. A geographic map analogy was used, in which large “heights” represent large distances in the feature space while low “valleys” represented subsets of similar data. Therefore, “mountain ranges” with "snow-covered" heights visually separate the clusters in the data [22]. ESOM/U-matrix is a projection technique from high dimensional data spaces onto two dimensions. The third direction added by the U-matrix is not an additional projection dimension, but the representation of the true distances of the data points in the projection plane. The cluster visualization for the ESOM/U-matrix projected data was further enhanced by calculating the U* (“U star”) matrix, which results from the combination of the U-matrix distances with the so-called P-matrix. The latter also uses the ESOM map as a floor plan, but instead of the local distances, density values in data space, measured by the weights of the neurons, are used as height values [7].

### 3.4. Additional Data Projection Techniques

Additional analyses were applied to data set #1, i.e., the structureless “golf ball” data set, to further determine that it actually has no structure at all. These analyses comprised firstly a principal component analysis (PCA), which was performed to project the three-dimensional data into a two-dimensional space of principal components (PCs). Secondly, autoencoding was applied, consisting of a multilayered network of artificial neurons.

Principal component analysis (PCA) uses the direction of the greatest variance in the high dimensional data space to reduce dimensions i.e., to project the data onto two dimensions [23]. This direction forms the first component (factor) of the projection. As a second component, an orthogonal direction to the first component is determined, along which the variance of the data is again maximum. The PCA was performed using the R library “FactoMineR” (https://cran.r-project.org/package=FactoMineR [8]).

Autoencoders use supervised learning multilayer feedforward artificial neuronal networks (ANN) to extract the essential features of the structure of a data set, which reduces its dimensions and can, therefore, be used for data projection. Autoencoders then learn to reconstruct the original data with the reduced representation. If the data set has a certain structure, this would be learned and emphasized in the reconstructed data. The training of an ANN is done with the goal of “identity”, i.e., all case vectors used as input to the autoencoder are reproduced identically as its output [24]. The neurons compute the logistic sigmoid function applied to the scalar product of the preceding neurons and the intermediate synoptical weights. As the learning method, backpropagation was used as a common implementation in autoencoders [25]. The network consisted of n = 3 input and n = 3 output neurons to represent the original “golf ball” data that are three-dimensional. Three hidden layers were used, each containing five, two, and five neurons. After training of the ANN to identity, the central two neurons represent a two-dimensional projection of the n-dimensional input data space, which is then used to reconstruct the data toward the three-dimensional output data space. The calculations were performed using the R library “ANN2” (https://cran.r-project.org/package=ANN2; [9]).

## 4. Conclusions

In this report, we have highlighted a possible pitfall of flow-cytometry related research due to data analysis-based biases owing to data transformation and projection. This may also be relevant for other molecular laboratory techniques of cell separation and tasks performed on comparable data. The increasing use of t-SNE in this area, supported by the increasing number of publications mentioning this method, may be subject to a revision. Alternative methods should be contemplated as a complement or replacement. However, the method of analysis is crucial to avoid non-reproducible pattern recognition, which may be due to an exaggeration of random outliers from otherwise more homogeneous data sets. In view of the present demonstrations, contemporary research methods focusing on the identification of subgroups in complex data sets may require review. It is essential that the data analysis algorithm correctly reflects the cluster structures in the data, which may not be guaranteed by widespread data projection methods. Therefore, we propose the ESOM/U*-matrix method as a viable unbiased alternative method to t-SNE. With default settings common in biomedical research units, it surpasses the uncritical use of t-SNE in the correct projection of structured biomedical data.
